# [Expression of Concern] Inhibition of tumorigenesis and invasion of hepatocellular carcinoma by siRNA-mediated silencing of the *livin* gene

**DOI:** 10.3892/mmr.2025.13683

**Published:** 2025-09-12

**Authors:** Hui Liu, Shaochuang Wang, Hangyong Sun, Zeya Pan, Weiping Zhou, Mengchao Wu

Mol Med Rep 3: 903–907, 2010; DOI: 10.3892/mmr.2010.355

Following the publication of this paper, it was drawn to the Editor's attention by a concerned reader that, regarding the cell invasion assay data shown in Fig. 4A, the ‘Control’ and ‘pU-siNC’ data panels appeared to share an overlapping section of data, such that data which were intended to show the results of differently performed experiments had apparently been derived from the same original source. The authors were contacted by the Editorial Office to offer an explanation for this apparent anomaly in the presentation of the data in this paper; however, up to this time, no response from them has been forthcoming. Owing to the fact that the Editorial Office has been made aware of potential issues surrounding the scientific integrity of this paper, we are issuing an Expression of Concern to notify readers of this potential problem while the Editorial Office continues to investigate this matter further.

